# Idiopathic Osteoporosis and Nephrolithiasis: Two Sides of the Same Coin?

**DOI:** 10.3390/ijms21218183

**Published:** 2020-10-31

**Authors:** Domenico Rendina, Gianpaolo De Filippo, Gabriella Iannuzzo, Veronica Abate, Pasquale Strazzullo, Alberto Falchetti

**Affiliations:** 1 Department of Clinical Medicine and Surgery, Federico II University, 80131 Naples, Italy; domenico.rendina@unina.it (D.R.); gabriella.iannuzzo@unina.it (G.I.); veronica.abate.1990@gmail.com (V.A.); strazzul@unina.it (P.S.); 2 Assistance Publique-Hôpitaux de Paris, Hôpital Robert Debré, Service d’Endocrinologie et Diabétologie Pédiatrique, 75019 Paris, France; gianpaolo.defilippo@aphp.fr; 3 French Clinical Research Group in Adolescent Medicine and Health, 75014 Paris, France; 4 Unit of Bone and Mineral Metabolic Diseases, Istituto Auxologico Italiano, San Giuseppe Hospital, Piancavallo, 28824 Verbania, Italy; 5 Istituto Auxologico Italiano, IRCCS, Unit for Bone Metabolism Diseases and Diabetes & Lab of Endocrine and Metabolic Research, 20145 Milan, Italy

**Keywords:** osteoporosis, nephrolithiasis, adult, children

## Abstract

Idiopathic osteoporosis and nephrolithiasis are formidable health problems showing a progressive increase in their incidence and prevalence in the last decades. These temporal trends were observed in both pediatric and adult populations worldwide. Epidemiological and experimental studies indicate that both disorders show several common pathogenic environmental and genetic factors. In this review, we analyzed the clinical characteristics common to the two disorders and the state-of-the-art knowledge regarding the genetic predisposition and the environmental factors recognized as triggers in adult and pediatric ages. As a result of this work, we propose to consider idiopathic nephrolithiasis and osteoporosis as two possible expressions of a unique clinical syndrome. Accordingly, the clinical approach to both disorders should be modified in order to program an efficient primary and secondary prevention strategy.

## 1. Introduction

Idiopathic osteoporosis and nephrolithiasis are formidable health problems characterized by high incidence and prevalence in the adult population worldwide [1]. The pathogenic mechanisms of these two diseases depend on the tight interaction between genetic and environmental factors. In this review, we will analyze the available knowledge regarding the genetic and environmental factors recognized as common triggers for both entities.

## 2. Definition and Epidemiological Data

Osteoporosis is defined as a decrease in bone density that results in micro-architecture deterioration, predisposing the affected patients to fractures [2]. Operationally, osteoporosis has been defined on the basis of a bone mineral density assessment using dual energy X-ray absorptiometry. According to the World Health Organization criteria, in adult subjects, osteoporosis is defined as a bone mineral density that lies 2.5 standard deviations or more below the average value for young healthy subjects (a T-score of < −2.5 standard deviations) [3]. This criterion has been widely accepted and provides both a diagnostic and an intervention threshold. Osteoporosis is recognized as the most common form of metabolic bone disease, with an estimated 200 million people affected worldwide; in particular, approximately 30% of postmenopausal women have osteoporosis in industrialized countries, including Europe and United States [4]. Great attention has been recently paid to pediatric osteoporosis and much has changed in the field of pediatric bone health since the first positions of the International Society for Clinical Densitometry were published in 2003 [5]. Some scientific societies have included bone health screening in their clinical guidelines for chronic childhood diseases [6]. Osteoporosis in childhood and adolescence is a condition characterized by a low bone mineral density or bone mineral content (z-score ≤ −2.0, adjusted for race, age and gender) and the presence of a clinically significant history of skeletal fractures [7]. In the absence of a history of fractures, unlike the T-score in adults, the z-score < −2 cannot be used for diagnosis of osteoporosis and thus the preferred term is reduced bone density according to chronological age [8]. Primary osteoporosis in children is a rare condition, recognizing a genetic background. More commonly, secondary osteoporosis (or, more frequently, reduced mineral density) can result from chronic diseases affecting mineral metabolism and/or from significant nutritional perturbations [9]. 

Nephrolithiasis refers to the presence of crystalline stones (calculi) within the urinary system (kidneys or ureters) [10]. It affects nearly 1 in 11 individuals in the United States at some point in their lives, and there is evidence that the number of those who have had a stone is rising [11]. Similar epidemiological data are available in Europe and in almost all industrialized countries [10,11,12]. Since the incidence of both osteoporosis and nephrolithiasis increases with age, it is expected that population aging worldwide will lead to a major increase in the incidence of both [1]. In pediatric patients, hospitalization for kidney stone disease has steadily increased [13]. In contrast to the adult kidney stone patient, in which dietary and metabolic factors play a major causal role, genetic-based metabolic disorders are the main causes of childhood nephrocalcinosis and urolithiasis: this notwithstanding, it is conceivable that some of the features of metabolic syndrome, in particular abdominal adiposity, may increasingly contribute to the pathogenesis of such a disorder in the pediatric population [13,14,15]. The incidence of urolithiasis in pediatric patients is estimated to be approximately one tenth of that in adults. Since the discovery of renal calculi is incidental in up to 40% of affected children, the real incidence in childhood is likely to be underestimated [14,15].

## 3. Natural History and Statistical Evidence

The natural histories of osteoporosis and nephrolithiasis have many similarities. Both disorders are charged with a high morbidity if not correctly diagnosed and accurately treated. Without a correct clinical and therapeutic approach, at least 40% of osteoporotic women and 15–30% of osteoporotic men will sustain one or more fragility fractures in their remaining lifetime [2]. Likewise, as shown by a meta-analysis of nine study cohorts involving 4,770,691 participants overall, stone-forming patients have a significant increased relative risk of chronic kidney disease and, in particular, of end-stage kidney disease compared to individuals without a history of nephrolithiasis [16].

What is much less known is that nephrolithiasis may be considered a risk factor for reduced bone mineral density and osteoporosis [17]. A recent meta-analysis of 24 case–control studies including 1595 patients with nephrolithiasis (590 women; mean age 41.1 ± 7.6 years) versus 3402 healthy controls (2109 women; mean age 40.2 ± 9.2 years) demonstrated that patients with nephrolithiasis showed significantly lower values of bone mineral density T-scores for the spine (7 studies; 390 patients with nephrolithiasis, 282 controls; standardized mean difference = −0.69; 95% confidence interval = −0.86 to −0.52; I2 = 0%; *p* < 0.05), total hip (seven studies; 521 patients with nephrolithiasis, 323 controls; standardized mean difference = −0.82; 95% confidence interval = −1.11 to −0.52; I2 = 72%; *p* < 0.05), and femoral neck (six studies; 360 patients with nephrolithiasis, 252 controls; standardized mean difference = −0.67; 95% confidence interval = −1.00 to −0.34; I2 = 69%; *p* < 0.05). In addition, two studies reported a higher prevalence of osteoporosis in patients with nephrolithiasis compared to controls (39,960 patients with osteoporosis, 79,800 controls; odds ratio = 4.12, 95% confidence interval 3.99 to 4.26, I2 = 0%, *p* < 0.05) [17]. However, the same meta-analysis gave conflicting results regarding the relationship occurring between nephrolithiasis and skeletal fractures. Four case–control studies demonstrated that patients with nephrolithiasis are at increased risk of fractures compared to controls (odds ratio = 1.15, 95% confidence interval = 1.12–1.17; *p* < 0.05), whereas two longitudinal studies did not confirm the association (hazard ratio = 1.31, 95% confidence interval = 0.95 to –1.62; I2 = 92%; *p* = 0.11) [17]. In a subsequent retrospective study involving calcium stone-forming patients, Sakhaee and colleagues found no significant association between urinary calcium excretion and femoral neck or lumbar spine bone mineral density in 250 men and in 145 postmenopausal women treated with estrogens. However, in 37 non-estrogen-treated postmenopausal women, lumbar spine bone mineral density correlated inversely with urinary calcium excretion during a calcium-restricted diet (r = −0.38, *p* = 0.04) and during fasting (r = −0.42, *p* = 0.05). These results confirm that nephrolithiasis is a risk factor for reduced bone mineral density and the occurrence of osteoporosis in post-menopausal women [18]. Fewer data are available about the influence of osteoporosis on the risk of nephrolithiasis incidence. Chou and colleagues, evaluating the incidence of symptomatic nephrolithiasis in a cohort of 1634 patients with newly diagnosed osteoporosis (239 men and 1395 women) and in a cohort of 6536 age- and sex- matched subjects without osteoporosis over a 5-year period, demonstrated that osteoporotic patients carry a higher risk of symptomatic nephrolithiasis (adjusted Hazard Ratio 1.38, 95% confidence interval 1.03 to 1.86; *p* < 0.05) [19]. According to Prochaska and colleagues, who carried out a prospective analysis of 96,092 women enrolled in the Nurses’ Health Study II, low bone density is an independent risk factor for incident nephrolithiasis (adjusted relative risk 1.39; 95% confidence interval 1.20 to 1.62, *p* < 0.05) and, among participants with low bone density, treatment with bisphosphonates was associated with a lower risk of kidney stones (adjusted relative risk 0.68; 95% confidence interval 0.48 to 0.98, *p* < 0.05). [20]. We have recently conducted an epidemiological survey involving 12,165 women and 629 men in Southern Italy (mean age 69.3 ± 10.0 years) of whom 10,157 had a clinical diagnosis of idiopathic osteoporosis, showing that osteoporosis is associated with an increased risk of nephrolithiasis (hazard ratio = 1.33, 95% confidence interval 1.01 to 1.74, *p* < 0.05) after adjustment for age, sex, body mass index, treatment for osteoporosis and smoking habits [21].

## 4. Pathogenesis 

**Figure 1:**
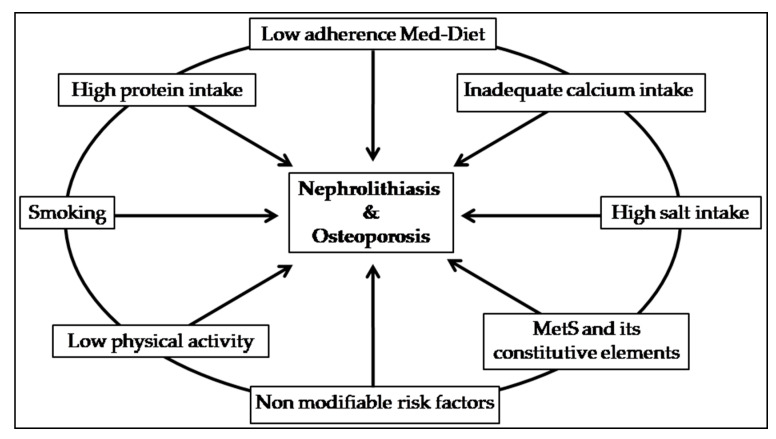
Common pathogenic factors between osteoporosis and nephrolithiasis. Med-Diet: Mediterranean Diet; MetS: Metabolic Syndrome. MetS constitutive elements: diabetes and/or high fasting plasma glucose, abdominal obesity, dyslipidemia and elevated blood pressure.

With regard to pathogenic mechanisms, idiopathic osteoporosis and nephrolithiasis are both multifactorial disorders and share common modifiable and non-modifiable causative factors (Figure 1) [2,10]. 

### 4.1. Modifiable Risk Factors

Diet. Among the modifiable risk factors, unhealthy eating habits involving high salt, high protein, high sugar and inadequate calcium intakes play a central role in the pathogenesis of both disorders [10,22]. Excessive salt intake has been focused on in the last few years as one of the main elements that influence health status [23]; in particular, high salt intake is linked to hypertension and its cardiovascular complications [24]. There is strong evidence of excessive salt intake starting in childhood and adolescence [25]. This is worth special attention, because the effect of the high salt intake will add a high amount of exposure time. Accordingly, many organizations have issued strong recommendations to reduce salt intake [26,27,28,29]. Moreover, the harmful relationship between salt intake, osteoporosis and nephrolithiasis is well established [30]. Bone is a target organ of a high dietary sodium intake [31]. Several experimental studies indicate that high salt intake increases urinary calcium excretion, a well-recognized risk factor for both osteoporosis and nephrolithiasis [32,33]. From a physiological point of view, in the renal proximal tubule, calcium handling is strongly dependent on sodium reabsorption [34]. High dietary salt intake induces a high-sodium load to the kidney and a condition of relative hypervolemia. According to the mechanisms of glomerular tubular balance, this condition diminishes the efficacy of sodium and water reabsorption in the proximal tubule [35]. As a consequence, the reabsorption of calcium, whose handling is passively coupled with sodium and water in this part of the nephron, will be less effective. Since, in the distal tubular sections, calcium reabsorption is unrelated to volume status, the final outcome will be a higher urinary calcium excretion [36]. On the other hand, a reduction in dietary salt intake induces relative hypovolemia, thus directly promoting sodium and indirectly calcium reabsorption in the proximal tubule, and subsequently lowering calciuria. The association between salt intake and calcium excretion was first reported by Kleeman and colleagues in 1964 [37]. These authors demonstrated a significant increase in calcium excretion upon the shift from a very-low-salt diet (1.1 g/day (19 mEq/day)) to a very-high-salt diet (24.5 g/day (419 mEq/day)). Since then, a large number of intervention studies have reported a linear association between salt intake and calcium excretion in healthy subjects, even for more modest levels of salt intake [38]. It has been suggested that an increase of 6 g/day (103 mEq/day) in salt intake may result in a 40 mg/day (1 mmol/day) increase in urinary calcium excretion [39]. Moreover, a 3.5 g (60 mEq/day) increase in salt intake leads to a 1.63-fold increase in the relative risk of hypercalciuria (defined as an urinary calcium excretion ≥7.5 mmol/24 h in men and ≥6.25 mmol/24 h in women), healthy subjects with a daily salt intake >10 g (171 mEq) having a 21.8% prevalence in hypercalciuria, compared with a 3.9% prevalence in those with a lower intake [40]. Other than urinary calcium excretion, a high salt intake is linked to a lower urinary excretion of citrate [41,42], one of the main inhibitors of lithogenesis that, in turn, significantly influences bone mineral mass [43]. Diets with a high salt content or a high content of animal protein impair citrate excretion also by inducing a subclinical intracellular and extracellular acidosis [44]. Hypocitraturia is a risk factor for nephrolithiasis also in children and adolescents: in a group of 493 Polish overweight/obese pediatric patients, the main factor predisposing patients to kidney stones was hypocitraturia [45]. Interestingly, in this group of patients, body mass index standard deviation score (BMI Z-score), total cholesterol, low-density lipoprotein cholesterol and triglycerides correlated negatively with citraturia, while high-density lipoprotein cholesterol correlated positively [45]. There is convincing evidence that increased salt and animal protein intakes are associated with an increased risk of nephrolithiasis [38,46]. This pathogenic link is more evident in stone-forming patients with metabolic syndrome, a significant risk factor for cardiovascular diseases, osteoporosis and nephrolithiasis [47,48]. In particular, in recurrent stone formers with metabolic syndrome, the urinary supersaturation index of calcium-oxalate salts (the most common component of renal calculi) is strictly dependent on the elevated dietary sodium intake and, in these patients, a reduction in sodium intake is associated with a substantial reduction in this index [49]. A recent meta-analysis of the available prospective studies demonstrated that higher dietary sodium intake was associated with significantly increased risk of osteoporosis, though a high degree of heterogeneity among studies was found [50]. Considered as a whole, the studies analyzing the association between salt intake and bone metabolism suggest that high dietary salt intake may be associated with impaired bone mineral content, reduced bone mineral density, and increased risk of osteoporosis; these relationships appear to be significantly influenced by anthropometric indices, ethnicity and gender [51]. Calcium intake is one of the main factors affecting the development of peak bone mass and preservation of bone mass in adults [52]. Epidemiological studies demonstrate a progressive reduction in dietary calcium intake with increasing age, resulting in a negative calcium balance [53,54]. This mechanism, in turn, significantly increases the risk of osteoporosis and calcium nephrolithiasis over time [55]. Calcium dietary intake is a key factor for oxalate absorption and excretion, oxalate being one of the most potent promoters of calcium nephrolithiasis [56]. A low calcium intake decreases the calcium concentration in the intestinal lumen and prevents the formation of insoluble and non-absorbable calcium-oxalate salts. Consequently, a higher quantity of soluble oxalate salts remains free in the intestinal lumen and can be easily absorbed and excreted by the kidney, increasing the urinary oxalate concentration, a significant metabolic risk factor for nephrolithiasis [57,58]. 

Moreover, a high dietary intake of simple sugars is another factor able to increase the risk of reduced bone mineral density and osteoporosis [59]. This increased risk has been attributed to the elevated urinary excretion of calcium and magnesium which occurs after the ingestion of common nutritive sugars such as glucose and sucrose [60], to the 1,25OH_2_D_3_ -dependent inhibition of intestinal and renal calcium transport, which occurs after a high dietary intake of fructose in experimental models [61], and to the impairment of bone formation caused by the reduced osteoblast proliferation, the increased osteoclast activation, and the increased lactic acid production which has observed in experimental models after high glucose dietary intake and has be linked to impairment of bone strength observed in type 1 and 2 diabetes [60,62,63]. In this regard, it should be noted that the regular consumption of soft drinks, a notoriously high source of added sugar, is strongly associated with an increased risk of developing fractures in children and adolescents [64,65,66,67] and nephrolithiasis, linked to high urinary calcium excretion, in adults [68,69].

With regard to dietary patterns, a Western type of diet, characterized by a significant share of highly processed and refined foods and high content of sugars, salt, fat and animal protein, has been recognized as an important factor contributing to the development of metabolic disorders such as osteoporosis and nephrolithiasis [70,71]. On the contrary, there is growing evidence that a higher adherence to a Mediterranean-type of diet significantly reduces the incidence of nephrolithiasis [72,73,74] and is associated with a reduced risk of bone fractures as well as with a higher mean bone mineral density [75,76]. The principles underlying the Mediterranean diet, recognized by UNESCO as an intangible cultural heritage of humanity [77], can be summarized as follows: high consumption of fruits, vegetables, cereals (especially whole seeds), legumes, and nuts; relatively high fat consumption (up to 40% of total energy intake), mainly as monounsaturated fatty acids from extra-virgin olive oil, the main fat used for seasoning and cooking; moderate to high consumption of fish; moderate dairy product consumption, usually in the form of yogurt and cheese; low red meat and meat product consumption; moderate alcohol consumption, mainly in the form of red wine during meals; low consumption of simple sugars (pastries, soft drinks, etc.); and high consumption of herbs and spices—an important source of micronutrients, including calcium—used to flavor dishes [78,79]. This dietary model has been shown to feature a significantly reduced level of oxidative stress thanks to its high antioxidant contents [80]. Oxidative stress occurs in fact as a result of an overproduction of oxygen free radicals not balanced by an adequate level of enzymatic and non-enzymatic antioxidants. It can damage cellular lipids, proteins, or DNA, inhibiting their physiological functions [81]. A high oxidative stress has been related to the pathogenesis of both osteoporosis and nephrolithiasis [82,83]. With regard to bone metabolism, reactive oxygen species directly influence both osteoblastogenesis and osteoclastogenesis. Their effects on these cell types are mediated by the same signaling cascades and factors, namely Extracellular signal-Regulated Kinase (ERKs), Nuclear Factor Kappa-light-chain-enhancer of activated B cells (NF-κB), Receptor Activator of Nuclear factor Kappa-Β Ligand (RANKL), Tumor Necrosing Factor (TNF), and interleukin-6, used by estrogens, however in the exact opposite manner [84,85]. In particular, reactive oxygen species impair osteoblastogenesis by at least two mechanisms: (1) antagonism of Wnt signaling; and (2) direct and sustained activation of ERKs and NF-κB [86]. On the other hand, reactive oxygen species inhibit osteoclast apoptosis and stimulate osteoclastogenesis by increasing RANKL production in cells of the stromal/osteoblastic lineage [87] as well as by an ERK/NF-κB/TNF/interleukin 6-mediated mechanism [88]. With regard to nephrolithiasis, experimental and clinical studies demonstrated that high oxidative stress, especially uncontrolled lipid peroxidation, occurs in patients with nephrolithiasis [89,90,91,92]. In a model of kidney stone formation pathway proposed by Yasui and colleagues, reactive oxygen species are produced during interactions between crystals and renal cells, and are responsible for various cellular responses. In particular, calcium oxalate monohydrate crystals attach to renal tubular cells, leading to the activation of cyclophilin D. Subsequently, mitochondrial collapse and oxidative stress, with production of reactive oxygen species, occur. These events activate apoptosis, cell injury and osteopontin expression. Thereafter, collapsed mitochondria and fragmented microvilli drop off into the urine and condense into kidney stones [93].

Low physical activity. An additional lifestyle risk factor for the occurrence of both osteoporosis and nephrolithiasis may be identified in physical inactivity, which was reported to be a risk factor for both disorders, at least in post-menopausal women [94,95]. In addition, reduced physical activity prevents the achievement of an optimal peak of bone mass in children and adolescents, thus predisposing patients to osteoporosis later in life [96]. On the contrary, a moderate and constant physical activity contributes to reducing the risk of forming kidney stones by reducing weight gain and the risk of type 2 diabetes and to preserve bone mineral content [97,98]. The cellular mechanotransduction is the biological process by which physical activity regulates bone homeostasis [99] with osteocytes playing a central role in this process [100]. The major mechanosensors are the osteocyte cytoskeleton, dendritic processes, integrin-based focal adhesions, connexin-based intercellular junctions, primary cilium, ion channels, and extracellular matrix [101]. Through them, the mechanical stimuli regulate numerous cellular functions, including gene expression, protein synthesis, cell proliferation and differentiation. The dysfunctions in mechanotransduction signaling or in the osteocyte response lead to an imbalance in bone homeostasis. This alteration is very relevant in some conditions such as osteoporosis and aging [102]. Interestingly, during long-duration spaceflight in absence of gravity-mediated mechanical stimuli on the skeleton, astronauts appear to be at increased risk of both osteoporosis and nephrolithiasis [103,104].

Smoking. Cigarette smoking is also a significant risk factor for both osteoporosis and nephrolithiasis [83,84]. Cigarette smoking predisposes patients to osteoporosis by different pathophysiologic mechanisms. In effect, cigarette smoking influences the metabolism of calciotropic, cortical and sexual hormones [105,106]. In addition, it directly induces alteration in the RANK—Receptor Activator of NF-kB Ligand (RANKL)—osteoprotegerin (OPG) system in collagen metabolism and in bone angiogenesis. Nicotine also has an inhibitory effect on osteogenesis [105,106].

On the other hand, two main mechanisms have been envisaged to explain the association between smoking and the increased risk of nephrolithiasis. First, smoking significantly increases plasma anti-diuretic hormone levels, thus decreasing urine volume and promoting urinary crystal supersaturation. The second mechanism is through increased production of reactive oxygen species resulting in increased oxidative stress and renal injury [105,107,108].

Primary and secondary preventive measures. Based on the available evidence, it is not surprising that nonpharmacological interventions for a correct primary and secondary prevention of nephrolithiasis and osteoporosis are similar. Both include the avoidance of weight gain and a reduction in overweight, the avoidance of smoking and alcohol abuse and regular practice of physical activity [97,98]. In addition, a normal calcium/low-sodium diet (1.2–1.5 g calcium/day and < 2 g sodium (or 5 g of salt)/day, respectively), with large consumption of vegetable proteins, and a water intake of at least 2 L per day, is recommended [10,98,109]. This same approach is a widely recognized valuable tool for the prevention of cardiovascular disease [110]. This type of intervention should be encouraged from childhood and adolescence. Regular physical activity in pre- and post-pubertal age is associated with the achievement of an optimal bone mass peak [111]. Twenty-five percent of adult bone mass is acquired at the time of pre and early puberty [111]. 

An adequate vitamin D status is fundamental for bone health [112,113,114]. However, considering the pivotal role of the vitamin D system in regulating calcium-phosphate homeostasis [112,113] and the high prevalence of vitamin D deficiency (i.e., serum 25-hydroxyvitamin D levels < 50 nmol/L or 20 ng/mL) worldwide and at all ages [113,114,115,116], the relationship between vitamin D status and susceptibility to nephrolithiasis ought to be carefully analyzed. Stone formers were found to have a high prevalence of vitamin D deficiency compared to non-stone formers [117]. Possible explanations for this association include secondary hyperparathyroidism and increased oxidative stress or inflammation in vitamin D deficiency [118]. Noteworthy, a Cochrane meta-analysis did not indicate an increased risk of hypercalcemia, hypercalciuria or nephrolithiasis in patients with vitamin D deficiency treated with cholecalciferol [119]. Moreover, the correction of vitamin D deficiency with cholecalciferol in patients with Paget’s disease of the bones, who are at increased risk of nephrolithiasis [120], did not cause either an increase in supersaturation indices of calcium-oxalate and calcium-phosphate salts or a rise in urinary pH and urinary excretion of urate, the major risk factors for calcium and urate nephrolithiasis, respectively [121].

### 4.2. Non-Modifiable Risk Factors

The familial association of both primary osteoporosis and idiopathic stone disease has been unraveled by numerous studies, although the specific genetic and epigenetic factors have remained less clear. Both disorders seem to be genetically heterogeneous diseases related to multiple genetic factors [122,123], identified on family-based or case–control studies (Table 1).

All humans carry many risk alleles for all common diseases, and each affected individual likely carries a higher burden and unique portfolio of risk variants. While the description of a polygenic model at the population level is very simply defined, it generates considerable genetic heterogeneity between individuals, which, in turn, is consistent with the characteristics of common complex diseases, such as heterogeneity in clinical presentation and variation in response to treatments. Understanding the consequences of polygenicity for individuals also links to an understanding of epistasis, the interacting effects of risk loci [143]. 

Calcium-sensing receptor (CASR). The calcium-sensing receptor is a plasma membrane G protein-coupled receptor that is expressed in the parathyroid hormone-producing chief cells of the parathyroid gland and in the cells lining the kidney tubule. In the parathyroid gland, the CASR senses small changes in circulating calcium concentration and couples this piece of information to intracellular signaling pathways that modify PTH secretion. In the kidney, the CaSR is expressed in all tubular segments and regulates tubular cell function in response to the increase in calcium concentrations in the interstitium or tubular lumen. In the proximal tubule, the CaSR inhibits PTH-induced phosphate excretion. In addition, CaSR inhibits calcium reabsorption in the medullary thick ascending limb and water reabsorption in the collecting duct. These CaSR-mediated effects ensue to dilute the tubular calcium load in a larger fluid volume and decrease the risk of salt precipitation, crystal aggregation and growth, leading, in the end, to stone formation and a reduced bone mineral density [124,125,126,144]. Natural polymorphic variants of CASR are associated to an increased risk of both nephrolithiasis and osteoporosis, directly influencing calcium tubular handling [124,125,126].

Vitamin D receptor (VDR). The vitamin D receptor is an intracellular hormone receptor that specifically binds 1,25(OH)_2_D_3_ and mediates its biological effects. VDR contains a zinc-finger DNA-binding and transcriptional activation domain and a ligand-binding domain [145]. Natural polymorphic variants of VDR are associated to an increased risk of both nephrolithiasis and osteoporosis, by directly affecting calcium and citrate tubular metabolism [127,128,129,130,131,132]. In addition, a natural polymorphic variant of VDR, which lacks only the first three amino acids, was shown to interact more efficiently with its transcription factor and to possess elevated transcriptional activity. This defect resulted in an increase in 1,25-dihydroxyvitamin D3 levels, hypocalcemia with secondary hyperparathyroidism and hypophosphatemia, leading to rickets. In addition, these patients also showed increased serum alkaline phosphatase levels, generalized aminoaciduria, total alopecia and, in some cases described in an Asian population, nephrolithiasis in adults and children [146]. 

Alkaline phosphatase (ALPL). Alkaline phosphatase are membrane-bound glycoproteins that hydrolyze various monophosphate esters at a high pH optimum. The enzyme acts physiologically as a lipid-anchored phosphoethanolamine and pyridoxal-5-prime-phosphate [147]. Allelic variants of the *ALPL* gene have been linked to an increased risk of both osteoporosis and nephrolithiasis [133,134]. In addition, twenty-three allelic variants have been isolated in children affected by hypophosphatasia and kidney stones. Every mutation disrupts the spatial relationship between two essential components of the ALP active pocket in the calcium binding domain [148].

Osteopontin (SPP1). Osteopontin is a multifunctional glycosylated phosphoprotein and is a member of the small integrin-binding ligand, N-linked glycoprotein (SIBLING) family. An analysis of gene expression using microarray technology has shown that the *SPP1* gene was markedly upregulated in rats during the development of calcium stone formation [149]. SPP1 seems to be involved in the early and in the late stages of the stone-forming process. In fact, that SPP1 plays a role in stimulating the deposition and adhesion of crystals to cells, due to increased adhesion tendency. Furthermore, many studies suggested that SPP1 is also an inhibitor of abnormal calcification and has a vital inhibitory role during crystallization, crystal retention, crystal congregation, and stone formation in vitro or in vivo [150,151]. Experimental studies also demonstrate that SPP1 plays a role in anchoring the osteoclasts on the bone mineral matrix, stimulated by calcitriol. In this condition, SPP1 accumulates along the bone surface and binds osteoclasts that allow local bone resorption [152]. Some polymorphic variants of *SPP1* gene are associated to an increased risk of both nephrolithiasis and osteoporosis, directly influencing calcium tubular handling [135,136].

Claudin-14 (CLDN14). Claudin-14 is an integral membrane protein and a component of tight junction strands, which regulates paracellular permeability at epithelial tight junctions, and its expression is regulated by extracellular calcium changes [153]. It has been reported to be associated with levels of urinary calcium and serum parathyroid hormone and may therefore regulate bone development through its regulatory effect on calcium metabolism. Transgenic overexpression of claudin-14 in mouse kidneys generated renal defects characterized by an uncontrolled loss of calcium and magnesium [153]. Some polymorphic variants of *CLDN14* gene are also associated to reduced bone mineral density in the hip and spine as well as to nephrolithiasis [137,138,139].

Fibroblast growth factor 23 (FGF23). Fibroblast growth factor 23 is a phosphaturic hormone whose physiological actions on renal tubule tissue are mediated by FGF receptors (FGFR) and klotho, which functions as a co-receptor, increasing the binding affinity of FGF23 for FGFRs. In the renal tubule, FGF23 regulates vitamin D metabolism and tubular phosphate reabsorption by modulating the metabolic activity of 1α 25OH Vitamin D Hydroxylase (Cyp27b1) and decreasing the tubular expression of type IIa sodium–phosphate cotransporter independently from PTH [154,155]. An excess of FGF23 serum levels is implicated in the pathogenesis of renal phosphate leak, a clinical disorder characterized by PTH- and vitamin D independent hypophosphatemia and reduced renal phosphate reabsorption [156,157]. This disorder predisposes patients to both osteoporosis and nephrolithiasis [141,156,157]. A functional allelic variant of the *FGF23* gene (*T239M, rs7955866*) has been described in stone-forming patients with renal phosphate leak. In vitro studies showed that the *T239M* change increases FGF23 secretion and that the *FGF23(239M)* variant induces a higher activation of the FGF receptor/ERK pathway compared to *FGF23(239T)* [142].

Type 2a sodium–phosphate cotransporter (*SLC34A1*). The type 2a sodium–phosphate cotransporter (NPT2a) is expressed in the apical membrane of renal proximal tubular cells and is a key-regulator of phosphate homeostasis, modulating urinary phosphate excretion [158]. In effect, phosphate filtered by the glomerulus is subsequently reabsorbed in the proximal tubule, in which the rate-limiting step is the uptake of phosphate through NPT2a [155,158]. Priè et al. identified two mutations in *SLC34A1* gene in two patients with a renal phosphate leak causing osteoporosis and nephrolithiasis [141]

Vitamin D 24-hydroxylase (CYP24A1). CYP24A1 is a mitochondrial enzyme responsible for inactivating vitamin D metabolites through the C-24 oxidation pathway. The 1,25-(OH)2D3 induces the 24-hydroxylase, whereas hypocalcemia, through increased parathyroid hormone, suppresses this enzyme. The mutant CYP24A1 enzymes revealed complete or near-complete loss of function, characterized by a weak binding of 1,25-dihydroxyvitamin D3 to 24-hydroxylase, leading, in the end, to hypercalcemia, nephrolithiasis and pseudovitamin D-deficient rickets [159,160]. A mutation of the *CYP24A1* gene has been reported in a 22-year-old male patient with recurrent nephrolithiasis, nephrocalcinosis, hypercalcemia, low parathyroid hormone levels, hypercalciuria and low bone mass [142].

## 5. Conclusions

As reported in previous sections, the bulk of epidemiological and experimental data support a statistical association between idiopathic nephrolithiasis and osteoporosis, two disorders sharing many similarities with regard to environmental and genetic background. Based on such evidence, we propose to consider idiopathic nephrolithiasis and osteoporosis as the possible expressions of a unique clinical syndrome. This view involves a holistic approach to the clinical management of patients with nephrolithiasis or osteoporosis and suggests the need to evaluate the consensual occurrence of either disorder in all affected patients. On practical grounds, this holistic approach involves the assessment of metabolic risk factors for nephrolithiasis (by the measurement of 24-h urinary excretion of calcium, phosphate, citrate, magnesium and urate) in patients with osteoporosis and, conversely, the evaluation of the bone mineral density by dual-energy X-ray absorptiometry in patients with a personal history of nephrolithiasis. In osteoporotic patients, an ultrasound examination of the abdomen should be also performed after the diagnosis of osteoporosis to evaluate the occurrence of kidney stones. This approach would guarantee the optimal treatment of most patients with either type of metabolic disorder. To this end, the development of a specific public health strategy is definitely warranted.

## Figures and Tables

**Table 1:** Genes associated to occurrence of osteoporosis and nephrolithiasis.

Gene	*HGNC Symbol*	*Location*	Ref.
*Calcium-sensing receptor*	*CASR*	*3q13.3-q21.1*	[124,125,126]
*Vitamin D receptor*	*VDR*	*12q13.11*	[127,128,129,130,131,132]
*Alkaline phosphatase*	*ALPL*	*1p36.12*	[133,134]
*Osteopontin*	*SPP1*	*4q22.1*	[135,136]
*Claudin 14*	*CLDN14*	*21q22.13*	[137,138,139]
*Type 2a sodium–phosphate cotransporter*	*SLC34A1*	*5q35.3*	[140]
*Fibroblast growth factor 23*	*FGF23*	*12p13.32*	[141]
*25(OH)D-* *24-hydroxylase*	*CYP24A1*	*20q13.2*	[142]

*HGNC:* Human Genome Organisation (HUGO) Gene Nomenclature Committee.
